# About the Special Kidney Issue

**DOI:** 10.24908/pocus.v7iKidney.15415

**Published:** 2022-02-01

**Authors:** Nathaniel Reisinger, Abhilash Koratala

**Affiliations:** 1 Division of Renal, Electrolyte and Hypertension, Perelman School of Medicine, Penn Medicine; 2 Division of Nephrology, Medical College of Wisconsin Wisconsin, Milwaukee USA

**Keywords:** kidney, renal, editor letter, special issue

We know what you’re thinking. we’ve heard it a thousand times: “Oh, you’re a kidney doctor who dinks around with ultrasound? What do you look for? Hydronephrosis?” You may be asking, “Is this issue just going to be a bunch of pictures of hydronephrosis and distended bladders?” And yes, for the thousandth time, in acute kidney injury it’s almost never wrong to get a kidney and bladder ultrasound as part of the initial workup.

But there’s so much more! Point of care ultrasound is an incredibly valuable tool to the nephrologist, not just for quickly assessing for urinary outflow obstruction, but for overall assessment of physiology, particularly for volume status assessment. Our raison d'être is building the evidence for point of care ultrasound in nephrology and to that end we present for your approval The POCUS Journal: Kidney Edition.

**Figure 1:**
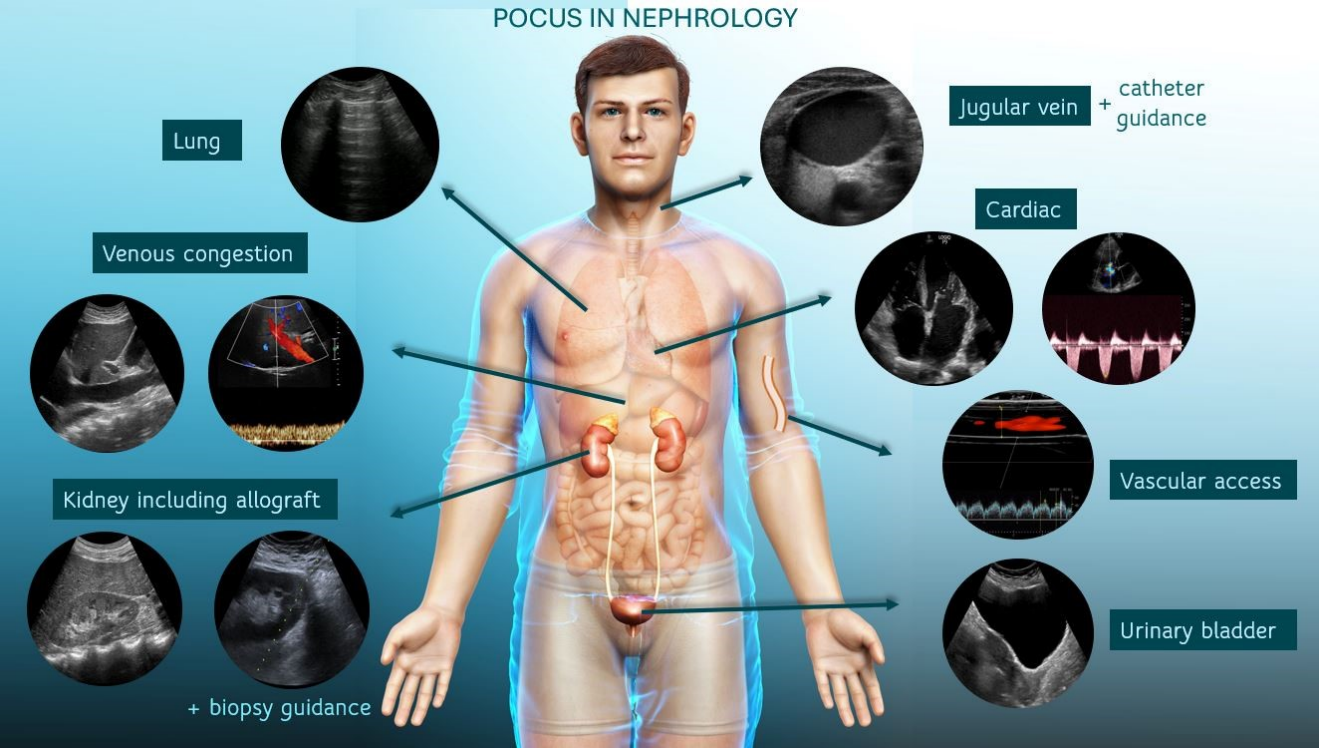
POCUS in Nephrology

As nephrologists, we see consults across an incredible range of clinical settings from the cardiac surgery ICU to the home dialysis clinic and from the glomerulonephritis clinic to the liquid oncology ward. Our scope of practice is likewise broad. We are consulted on electrolyte disorders, acid-base issues, mineral metabolism concerns, and hypertension but we also see patients with acute kidney injury and chronic kidney disease as well as any patient needing dialysis including intermittent, continuous, and peritoneal. We are primary care providers for those with CKD and critical care physicians for those needing CRRT. 

In all of these patients: those with hypertension, hyponatremia, metabolic alkalosis, acute kidney injury, chronic kidney disease, and dialysis dependence we are constantly asking: what is the volume status? Will the patient improve with diuretics in the cardiorenal syndrome? Will this hypovolemic patient with AKI respond to a crystalloid bolus? How much ultrafiltration do I prescribe for this routine hemodialysis session? As you will read in the ensuing pages, point of care ultrasound helps answer these questions. 

Moreover, we are most often consulted in situations where the kidney problem is not the overarching concern, but rather the first end-organ manifestation of a potentially life-threatening or emergent systemic problem. Sometimes this is obvious like in a patient rigoring with septic shock with a lactate of 10 on his way to the MICU or the patient who spent 4 hours on cardiopulmonary bypass with a prolonged aortic crossclamp time following a complex cardiac surgery. Other times, the cause of the fall in GFR is cryptic and transient or subtle like a hemodynamic rise in creatinine in a patient on combination of RAAS blockers, diuretics, and NSAIDs or a patient with mild tubular injury due to failure of autoregulation related to transient hypotension during induction anesthesia. 

One of our first experiences with point of care ultrasound is illustrative. A 45-year-old man with heart failure and a reduced ejection fraction due to non-ischemic cardiomyopathy was admitted for heart failure optimization in the coronary care unit. He had had a pacemaker placed on Friday and transferred to the CCU on Monday with acute kidney injury with creatinine rising from 1 to 3 over the weekend. Our signout noted his blood pressures were “soft” and his physical exam was “difficult.” Nephrology had been consulted over the weekend and proposed workup with urinalysis and kidney ultrasound was pending. Our physical exam was indeed challenging. His jugular veins were obscured due to his body habitus and his heart sounds were muffled. He was altered and could not hold his breath to assess for pulsus paradoxus. Ultimately our team performed a bedside echocardiogram which revealed a large pericardial effusion and tamponade physiology. He went to the OR within hours for a pericardial window and had 1 liter of blood drained with resolution of the hypotension and acute kidney injury post-operatively—having had cardiac tamponade complicating pacemaker placement.

In retrospect what strikes me most about this case—apart from the excellent demonstration of the utility of POCUS—is the proximity of the nephrologist to the front line of care in emergent situations. A nephrologist equipped with POCUS would have made the diagnosis 48 hours earlier, avoiding potential lasting harm by mitigating the extent of organ injury. Our experience as practicing nephrologists is exactly that: POCUS expedites care and adds value, not just for routine matters of exclusion of urinary obstruction, but overall integrative patient assessment.

We planned and executed this issue of The POCUS Journal as an entry point for nephrologists interested in getting started with point of care ultrasound and for providers from other disciplines to get a sense of what it’s like to be a nephrologist and gain some perspective on cases we see. For this issue, we are delighted to collaborate with the Renal Fellow Network—an online community of free open-access online medical education geared toward nephrology fellows. We will be publishing blog post commentaries simultaneously with the release of this issue to broaden our impact and reach more trainees. 

In this issue, you’ll find a panoply of articles on point of care ultrasound in nephrology. We start with two original research pieces on educating the next generation of nephrologists. We move on to case files and reports ranging from the mundane—an unusual case of unexpected obstructive nephropathy—to the sublime—integrating focused cardiac ultrasound and venous excess ultrasound into our daily rounds. We wrap up with reviews on a diverse range of topics including established topics like kidney ultrasound, but also practice-changing POCUS breakthroughs such as quantitative lung ultrasound and VExUS.

Finally, we’d like to thank the huge community of nephrologist- and non-nephrologist-POCUS enthusiasts who contributed to this issue. First we’d like to thank all of our outstanding fellows who submitted excellent cases. We’d like to thank all of our authors and reviewers who volunteered, taking the time out of their busy lives. We'd especially like to thank editor-in-chief Jamie Galen and former editor-in-chief Amer Johri for trusting us with this amazing opportunity, surrendering their journal to subspecialists for a month. Finally, we would be remiss if we did not thank managing editor Julia Herr. Without her organization, diligence, and hard work this issue could not have happened. 

Nathaniel Reisinger, MD

Abhilash Koratala, MD

